# Supplementary material for: A New Method for the Assessment of Myalgia in Interstitial Lung Disease: Association with Positivity for Myositis-Specific and Myositis-Associated Antibodies
Source: Diagnostics (Basel). 2022 May 4;12(5):1139. doi: 10.3390/diagnostics12051139 (PMC9140063; doi:10.3390/diagnostics12051139)
Supplement: Supplementary file 1 [file diagnostics-12-01139-s001.zip › diagnostics-1689277-supplementary.pdf]

Table S1. Autoantibodies in the three groups studied.

**Antibodies**	**Group A (M+ILD+) n.63**	**Group B (M-ILD+) n.104**	**Group C (M+ILD-) n.174**	**A vs B**	**B vs C**	**A vs C**
RF	7.9%	11.5%	2.3%	**0.05**<br>**X^2= 0.5**<br>**95CI= -0.9-12.4**	**0.001**<br>**X^2= 10.1**<br>**95CI= 3.3-16.9**	**0.05**<br>**X^2= 4**<br>**95CI= 0-15**
ACPA	3.2%	9.6%	1.2%	n.s.	**0.0009**<br>**X^2= 11**<br>**95CI= 3.2-15.6**	n.s.
ANA*	68.2%	52.9%	22.4%	n.s.	**<0.0001**<br>**X^2= 27**<br>**95CI= 18.9-41.3**	**<0.0001**<br>**X^2= 42.7**<br>**95CI= 31.8-57.4**
Nucleolar	14.3%	10.6%	1.2%	n.s.	**0.0004**<br>**X^2= 12.7**<br>**95CI= 3.9-16.8**	**<0.0001**<br>**X^2= 17.7**<br>**95CI= 5.9-23.8**
Speckled	42.9%	25%	24.1%	**0.01**<br>**X^2=5.8**<br>**95CI= 3.2-32.2**	n.s.	**0.005**<br>**X^2=7.9**<br>**95CI= 5.4-32.4**
Cytoplasmic	12.7%	19.2%	6.3%	n.s.	**0.001**<br>**X^2= 10.9**<br>**95CI= 5-21.9**	n.s.
Centromeric	3.2%	0%	0%	n.s.	n.s.	n.s.
Homogeneous	1.5%	8.7%	0.6%	n.s.	**0.0005**<br>**X^2= 12.2**<br>**95CI= 3.3-15.1**	n.s.
Anti-Ro60k	6.4%	8.7%	1.2%	n.s.	**0.002**<br>**X^2= 9.5**<br>**95CI= 2.5-14.5**	**0.02**<br>**X^2= 5**<br>**95CI= 0-14**
Anti-La	3.2%	2.9%	0%	n.s.	**0.02**<br>**X^2= 5.1**<br>**95CI= 0-8.2**	**0.01**<br>**X^2= 5.6**<br>**95CI= 0-10.9**
Anti-Sm	6.4%	7%	0%	n.s.	n.s.	**0.0008**

						**X^2= 11.3**<br>**95CI= 2-15.3**
Anti-Scl70	3.2%	2.9%	0%	n.s.	**0.02**<br>**X^2= 5.1**<br>**95CI= 0-8.2**	**0.01**<br>**X^2= 5.6**<br>**95CI= 0-10.9**
Anti-DsDNA	3.2%	1.9%	0.6%	n.s.	n.s.	n.s.
Anti Histone	0%	1.9%	0.6%	n.s.	n.s.	n.s.
ANCA	1.5%	2.9%	0%	n.s.	**0.02**<br>**X^2= 5.1**<br>**95CI= 0-8.2**	n.s.
Anti-Jo1	11.1%	8.7%	0.6%	n.s.	**0.0005**<br>**X^2= 12.2**<br>**95CI= 3.3-15.1**	**0.0001**<br>**X^2= 15.5**<br>**95CI= 4.3-20.6**
Anti-PL7	9.5%	3.8%	1.8%	n.s.	n.s.	**0.006**<br>**X^2= 7.4**<br>**95CI= 1.7-17.5**
Anti-PL12	4.8%	1.9%	1.8%	n.s.	n.s.	n.s.
Anti-OJ	3.2%	1%	0.6%	n.s.	n.s.	n.s.
Anti MDA5	4.8%	1%	1.8%	n.s.	n.s.	n.s.
Anti-Tif1γ	0%	1%	1.2%	n.s.	n.s.	n.s.
Anti-SAE1	1.5%	0%	0.6%	n.s.	n.s.	n.s.
Anti-Mi2	3.2%	3.8%	4.6%	n.s.	n.s.	n.s.
Anti-SRP	1.5%	2.9%	1.8%	n.s.	n.s.	n.s.
Anti-RNP	8.2%	1.9%	2.3%	n.s.	n.s.	**0.04**<br>**X^2= 4.3**<br>**95CI= 0.1-15.4**
Anti-PM/Scl	12.7%	9.6%	3.4%	n.s.	n.s.	**0.007**<br>**X^2= 7.3**<br>**95CI= 2.1-19.9**
Anti-Ro52k	28.6%	25%	1.8%	n.s.	**<0.0001**<br>**X^2= 37.2**<br>**95CI= 15.2-32.4**	**<0.0001**<br>**X^2= 40.7**<br>**95CI= 16.6-40**
Anti-Ku	4.8%	3.8%	1.2%	n.s.	n.s.	n.s.

Legend: ACPA: Anti Citrullinated Protein Antibody; ANCA: Anti Neutrophil Cytoplasmic Antibody; ANA: Antinuclear Antibody; ILD: interstitial Lung Disease; M: Myalgia; n.s.: not significant; RF: Rheumatoid Factor; *= Combined pattern of ANA were: M+ILD+ 3 (2 speckled-cytoplasmic; 1 nucleolar-cytoplasmic); M-ILD+ 10 (4 speckled-cytoplasmic; 3 nucleolar-cytoplasmic; 3 homogenous-cytoplasmic); M+ILD- 1 (speckled-cytoplasmic). Not reported antibodies were not found in any of the three groups.

Table S2. Patients with Multiple MSA/MAA positivity.

Group	MSA	MAA
M+ILD+	Anti-SAE1, Anti-MDA5	Anti-Ro52Kd
M+ILD+	Anti-PL7, Anti-SRP, Anti-Mi2	Anti-Ku
M+ILD+	Anti-PL12	Anti-Ro52Kd
M+ILD+		Anti-RNP, Anti-Ro52Kd
M+ILD+	Anti-PL7	Anti-Ku
M+ILD+	Anti-PL7	Anti-RNP
M+ILD+	Anti-PL12	Anti-Pm/scl
M+ILD+	Anti-Jo1	Anti-RNP
M+ILD+	Anti-Jo1	Anti-Ro52Kd
M+ILD+	Anti-Jo1	Anti-Ro52Kd
M+ILD+	Anti-Jo1	Anti-Ro52Kd
M+ILD+	Anti-Jo1	Anti-Ro52Kd
M-ILD+	Anti-PL7	Anti-Ro52Kd
M-ILD+	Anti-PL12	Anti-Ro52Kd
M-ILD+	Anti-Jo1	Anti-Ro52Kd
M-ILD+	Anti-Jo1	Anti-Ro52Kd
M-ILD+	Anti-Jo1	Anti-Ro52Kd
M-ILD+	Anti-Jo1	Anti-Ro52Kd
M-ILD+	Anti-Jo1	Anti-Ro52Kd
M-ILD+		Anti-Pm/scl, anti-Ro52Kd
M-ILD+		Anti-Pm/scl, anti-RNP
M-ILD+	Anti-PL7, Anti-SRP	
M-ILD+	Anti-PL12, Anti-Mi2	Anti-Ku, anti-Pm/scl
M-ILD+	Anti-OJ, Anti-Mi2	Anti-Ro52Kd
M+ILD-		Anti-pm/scl, anti RNP
M+ILD-	Anti-Mi2, anti-SAE1	Anti-Pm/scl
M+ILD-	Anti-PL7,anti-Tif1 γ, anti-Mi2	
M+ILD-	Anti-PL7, antiPL12, anti-SRP	
M+ILD-	Anti-PL7, anti-Mi2	
M+ILD-	Anti-OJ	Anti-Pm/scl

M+ILD-	Anti-Mi2, anti-SRP	

Legend: ILD: Interstitial Lung Disease; M: Myalgia.

Table S3. Association between the items included in the patients' selection.

	M1	TS	W	ME	dys	F	IIM	CTD	A	PMR	RP	PF	T	Skin	M	Fe	sicca	UIP	NSIP	OP	NVC+	BCs	HC	ANA	cyt	nucl	spec	hom	ACA
**M1**	//	1	3	3	3	0	2	0	0	3	0	1	1	1	0	0	1	0	0	0	0	0	0	0	0	0	0	0	0
**TS**	1	//	0	0	0	0	2	1	0	0	0	2	2	3	0	0	0	0	0	0	2	0	0	1	0	0	0	0	0
**W**	3	0	//	3	3	0	3	2	0	3	0	0	0	0	0	2	0	0	0	0	0	0	0	0	0	0	1	0	0
**ME**	3	0	3	//	0	0	3	0	0	0	0	0	0	0	0	2	2	2	0	0	0	0	0	0	0	0	0	0	0
**Dys**	3	0	3	0	//	2	2	2	0	0	0	2	2	0	0	0	0	0	0	0	1	0	0	0	0	1	0	0	0
**F**	0	0	0	0	2	//	1	2	0	0	0	1	2	0	3	0	0	0	0	0	1	0	0	1	0	0	0	0	0
**IIM**	2	2	3	3	2	1	//	3	1	0	2	1	2	2	1	1	0	0	0	0	0	0	0	0	0	1	0	0	0
**CTD**	0	1	2	0	2	2	3	//	3	0	2	2	2	0	2	1	0	0	0	0	1	0	0	1	0	0	0	0	0
**A**	0	0	0	0	0	0	1	3	//	0	1	1	0	0	0	0	0	0	0	0	0	0	0	0	0	0	0	0	0
**PMR**	3	0	3	0	0	0	2	0	0	//	0	0	0	0	1	0	0	0	0	0	0	0	0	1	0	1	0	0	0
**RP**	0	0	0	0	0	0	0	2	1	0	//	3	3	1	1	0	0	0	0	0	1	0	0	0	0	0	0	0	0
**PF**	1	2	0	0	2	1	1	2	1	0	3	//	3	1	0	1	0	0	0	0	3	0	0	3	0	1	0	0	0
**T**	1	2	0	0	2	2	2	2	0	0	3	3	/	2	0	0	0	0	0	0	2	0	0	2	0	3	0	0	0
**Skin**	1	3	0	0	0	0	2	0	0	0	1	1	2	//	1	0	0	0	0	0	2	0	0	1	0	0	0	0	0
**M**	0	0	0	0	0	3	1	2	0	1	1	0	0	1	//	0	0	0	0	0	0	0	0	0	0	0	1	0	0
**Fe**	0	0	2	2	0	0	1	1	0	0	0	1	0	0	0	//	0	0	0	1	0	0	0	0	0	0	0	0	0
**Sicca**	1	0	0	2	0	0	0	0	0	0	0	0	0	0	0	0	//	0	0	0	0	0	0	0	0	0	0	0	0
**UIP**	0	0	0	2	0	0	0	0	0	0	0	0	0	0	0	0	0	//	//	//	0	0	1	0	0	0	0	0	1
**NSIP**	0	0	0	0	0	0	0	0	0	0	0	0	0	0	0	0	0	//	//	//	0	0	0	0	0	0	0	0	0
**OP**	0	0	0	0	0	0	0	0	0	0	0	0	0	0	0	1	0	//	//	//	0	0	0	0	0	0	0	0	0
**NVC+**	0	2	0	0	1	1	0	1	0	0	1	3	2	2	0	0	0	0	0	0	//	3	0	0	0	0	0	0	0
**BCs**	0	0	0	0	0	0	0	0	0	0	0	0	0	0	0	0	0	0	0	0	3	//	0	0	0	0	0	0	0
**HC**	0	0	0	0	0	0	0	0	0	0	0	0	0	0	0	0	0	1	0	0	0	0	//	0	0	0	0	0	0
**ANA**	0	1	0	0	0	1	0	1	0	0	0	3	2	1	0	0	0	0	0	0	0	0	0	//	//	//	//	//	//
**Cyt**	0	0	0	0	0	0	0	0	0	1	0	0	0	0	0	0	0	0	0	0	0	0	0	//	//	//	//	//	//
**Nucl**	0	0	0	0	1	0	1	0	0	0	0	1	3	0	0	0	0	0	0	0	0	0	0	//	//	//	//	//	//
**Spec**	0	0	1	0	0	0	0	0	0	1	0	0	0	0	1	0	0	0	0	0	0	0	0	//	//	//	//	//	//
**Hom**	0	0	0	0	0	0	0	0	0	0	0	0	0	0	0	0	0	0	0	0	0	0	0	//	//	//	//	//	//
**ACA**	0	0	0	0	0	0	0	0	0	0	0	0	0	0	0	0	0	1	0	0	0	0	0	//	//	//	//	//	//

Legend: A: arthritis; ACA: anticentromeric pattern of ANA; ANA: Antinuclear antibodies; BC: Bushy Capillaries; cyt: cytoplasmic pattern; CTD: final diagnosis of Connective Tissue Disease; Dys: dysphagia; F: female; Fe: Fever; HC: history of cancer; homo: homogeneous pattern of ANA;IIM: Final Diagnosis of Idiopathic Inflammatory Myopathy; M: myalgia; ME: muscular enzymes; M1: at least 1 from proximal weakness, dysphagia, increased level of transaminases, Creatine phosphokinase, Lactic Dehydrogense; or TS; RP: PMR: Polymyalgia Rheumatica; PF: puffy fingers; Raynaud's Phenomenon; NSIP: Nonspecific Interstitial Pneumonia; nucl: nucleolar pattern of ANA; NVC: Nailfold Videocapillaroscopy; OP: Organizing Pneumonia; Spec: speckled pattern of ANA; Skin: TS+MH and other skin sign of IIM; T: telangiectasias TS: Typical skin rashes (Heliotrope Rash, Gottron's papules/sign); w: proximal weakness; UIP: Usual Interstitial Pneumonia; 0= not significant. 1=<0.05; 2=<0.01;3==<0.0001.

Table S4. Diagnoses in ILD patients with increased level of muscular enzymes.

Patients (gender, age)	Enzymes	Myalgia	Diagnosis
F 82	Myoglobin 84/55 ng/ml	Yes	IPAF
F 27	Aldolase 13 U/L	No	SSc+DM
F 55	LDH 848 U/L	No	iNSIP
M 69	LDH 624 U/L	No	iNSIP
M 82	LDH 615 U/L	No	IPAF
F 67	LDH 600 U/L	Yes	IPF
M 64	LDH581 U/L	No	IPAF
M 72	LDH 558 U/L	No	iNSIP
F 76	LDH 555 U/L, CPK 278 U/L	Yes	pSS
F 84	LDH 460 U/L	No	IPF
M 46	LDH 450 U/L	No	iNSIP
M 74	LDH 354 U/L, CPK 256 U/L	No	PM
F 57	LDH 300 U/L	No	iNSIP
M 65	LDH 299 U/L	No	IPF
F 68	LDH 282 U/L	No	IPAF
F 70	LDH 275 U/L	No	IPAF
F 80	LDH 263 U/L, CPK 281 U/L	Yes	AS
M 60	LDH 260 U/L	No	IPF
M 70	CPK 350 U/L	Yes	AS
F 68	CPK 283 U/L	No	AS
F 56	CPK 280 U/L	No	AS
M 63	CPK 183 U/L	No	AS
F 42	CPK 1501 U/L	Yes	PM
F 48	CPK 1290 U/L	Yes	AS
F 64	CPK 912 U/L	No	IPAF
F 68	CPK 850 U/L	Yes	SLE+PM
F 38	CPK 600 U/L	No	SSc+PM
F 71	CPK 560 U/L	No	PM

M 66	CPK 499 U/L	No	SSC+PM
M 48	CPK 383 U/L	Yes	AS
F 59	CPK 379 U/L	No	AS
F 48	CPK 351 U/L	No	AS
F 44	CPK 3412 U/L	Yes	AS
M 71	CPK 320 U/L	No	RA
F 50	CPK 283 U/L	No	AS
M 50	CPK 270 U/L	No	PM
M 43	CPK 275 U/L	No	PM
M 67	CPK 250 U/L	No	IPAF
M 67	CPK 245 U/L	No	IPF
M 61	CPK 235 U/L	No	IPF
M 65	CPK 224 U/L	No	RA
F 64	CPK 1992 U/L	Yes	pSS
F 57	CPK 184 U/L	No	IPAF
F 44	CPK 1379 U/L	Yes	SSc+DM
F 67	CPK 1280 U/L	Yes	IPAF

Legend: AS: Antisynthetase Syndrome; CPK: Creatine Phosphokinase; DM: Dermatomyositis; F: Female; IPAF: Interstitial Pneumonia with Autoimmune Features; IPF: Idiopathic Pulmonary Fibrosis; iNSIP: Idiopathic Nonspecific Pneumonia; M: Male; PM: Polymyositis; pSS: Primary Sjogren’s Syndrome; RA: Rheumatoid Arthritis; SLE: Systemic Lupus Erythematosus SSc: Systemic Sclerosis.

Table S5. Diagnoses in ILD patients.

**Diagnosis**	**M+ILD+ 63 patients**	**M-ILD+ 104 patients**	**p**
IPAF	23.8%	28.8%	n.s.
AS	27%	15.4%	n.s.
pSS	14.3%	10.6%	n.s.
DM/PM	9.5%	3.8%	n.s.
SSc	4.8%	1%	n.s.
MCTD	1.6%	0%	n.s.
SLE	1.6%	0%	n.s.
RA	3.2%	3.8%	n.s.
OS myositis	4.8%	4.8%	n.s.
Other OSs	0%	2.9%	n.s.
MPA	0%	1%	n.s.
IPF	4.8%	14.4%	n.s.
HP	1.6%	1%	n.s.
iNSIP	3.2%	6.7%	n.s.
COP	0%	3.8%	n.s.
iUIPp	0%	1.9%	n.s.

Legend: AS: Antisynthetase Syndrome; COP: Cryptogenic Organizing Pneumonia; DM: Dermatomysitis; HP: Hypersensitivity Pneumonia; iNSIP: idiopathic Nonspecific Interstitial Pneumonia; iUIPp: idiopathic probable Usual Interstitial Pneumonia ILD: Interstitial Lung Disease; IPAF: Interstitial Pneumonia with Autoimmune Features; IPF: Idiopathic Pulmonary Fibrosis; M: Myalgia; MCTD: Mixed Connective Tissue Disease; MPA: Micro Polyangiitis; n.s.: not significant; OS: Overlap Syndrome; PM: Polymyositis; pSS: Primary Sjögren's Syndrome; RA: Rheumatoid Arthritis; SLE: Systemic Lupus Erythematosus; SSc: Systemic Sclerosis. OS were the following M+ILD+ 3 (SSc+DM, SLE+PM, AS+SS), M-ILD+ 8 (SSc+PM 2, SSc+DM 2, SSc+RA 2, SLE+RA, RA+SS).
